# Insight into Biological Apatite: Physiochemical Properties and Preparation Approaches

**DOI:** 10.1155/2013/929748

**Published:** 2013-09-01

**Authors:** Quan Liu, Shishu Huang, Jukka Pekka Matinlinna, Zhuofan Chen, Haobo Pan

**Affiliations:** ^1^Dental Materials Science, Faculty of Dentistry, The University of Hong Kong, Hong Kong; ^2^Department of Orthopedics and Traumatology, The University of Hong Kong, Hong Kong; ^3^Department of Oral Implantology, Hospital of Stomatology, Guanghua School of Stomatology, Institute of Stomatological Research, Guangdong Provincial Key Laboratory of Stomatology, Sun Yat-sen University, Guangzhou 510080, China; ^4^Center for Human Tissues and Organs Degeneration, Shenzhen Institute of Advanced Technology, Chinese Academy of Sciences, Shenzhen 518055, China

## Abstract

Biological apatite is an inorganic calcium phosphate salt in apatite form and nano size with a biological derivation. It is also the main inorganic component of biological hard tissues such as bones and teeth of vertebrates. Consequently, biological apatite has a wide application in dentistry and orthopedics by using as dental fillers and bone substitutes for bone reconstruction and regeneration. Given this, it is of great significance to obtain a comprehensive understanding of its physiochemical and biological properties. However, upon the previous studies, inconsistent and inadequate data of such basic properties as the morphology, crystal size, chemical compositions, and solubility of biological apatite were reported. This may be ascribed to the differences in the source of raw materials that biological apatite are made from, as well as the effect of the preparation approaches. Hence, this paper is to provide some insights rather than a thorough review of the physiochemical properties as well as the advantages and drawbacks of various preparation methods of biological apatite.

## 1. Introduction

Biological apatite is an inorganic calcium phosphate salt (CaPs) in apatite form with a biological derivation. As the main inorganic component of both bones and teeth, it is distributed in the organic constituents with certain sequence and direction [1]. By serving as the fillers in such biological tissues, biological apatite is critical to the physicochemical properties of the bulk materials. Meanwhile, it acts as the main mineral storage of vertebrates, participating in the dissolution and precipitation processes of CaPs, as well as the absorption and formation of bones, dentine, and cementum* in vivo *[2]. Additionally, due to the similarity in chemical compositions and structure, together with its outstanding bioactivity and biocompatibility, biological apatite has been used as bone substitutes for the reconstruction of bone defect in oral implantology, periodontology, oral, and maxillofacial surgery as well as orthopedics [3, 4]. Given the significant role of biological apatite in the structure and function of biological tissues and its clinical applications, numerous studies have been carried out in the investigation of its basic physiochemical and biological properties [5–19]. On one hand, with different approaches, biological apatite with varied size, shape, chemical composition, and solubility was obtained in these studies. On the other hand, in all these academic and clinical investigations, the preparation process of biological apatite is usually the first stage, which may probably have great influence on the observation and assessment of the resultant's characteristics. Given all above, this paper is to give some insight into the physiochemical properties and the preparation approaches of biological apatite. 

## 2. Basic Characteristics of Biological Apatite

### 2.1. Chemical Compositions

From a chemical viewpoint, biological apatite could be regarded as a ramification of hydroxyapatite (HAp), that is, calcium phosphate, in apatite form. As a representative member of apatites, HAp plays a significant role in various fields, such as soil chemistry [21], dental materials [22], caries etiology [23], bone mineralization [24], osteoporosis [25], and drug delivery [26]. As the precursor of biological apatite, the composition and structure of synthetic HAp have been studied extensively in previous reports [27–29]. Two different crystal forms were reported, that is, hexagonal with the lattice parameters *a* = *b* = 9.432 Å, *c* = 6.881 Å, and *γ* = 120° [27] and monoclinic with the lattice parameters *a* = 9.421 Å, *b* = 2*a*, *c* = 6.881 Å, and *γ* = 120° [28]. These two forms share the same elements, with a stoichiometric Ca/P ratio of 1.67. The major difference in their structure is the orientation of hydroxyl groups. In the hexagonal HAp, two adjacent hydroxyl groups point at the reverse direction; while in the monoclinic form, hydroxyl groups have the same direction in the same column and an opposite direction among columns [30].

**Figure 1:**
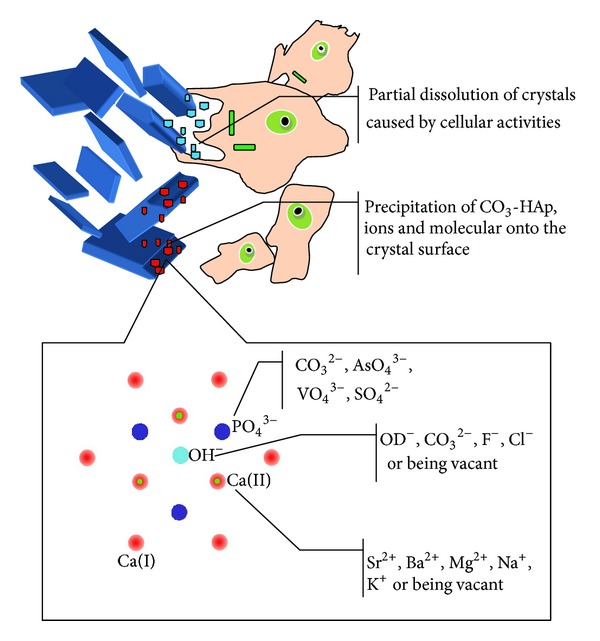
Schematic drawing of partial dissolution/precipitation of biological apatite *in vivo* and ionic substitutions in the crystal of HAp.

The structure of apatite allows for wide compositional variations because of its ability in detaining different ions in its three sublattices [31]. In detail, the site of Ca^2+^ may be occupied by bivalent or monovalent cations such as Sr^2+^, Ba^2+^, Mg^2+^, Na^+^, and K^+^, whereas P could be substituted by atoms such as C, As, V, S, while hydroxyl (OH^−^) may be replaced by OD^−^, CO_3_^2−^, F^−^, Cl^−^ or even be left vacant [31] (Figure 1). Given this, the apatite may host carbonate in two positions: the hydroxyl sublattice producing type A carbonate apatite (CO_3_-HAp) and the phosphate (PO_4_^3−^) sublattice forming type B CO_3_-HAp [31]. In physiological environment, partial dissolution of CaPs occurs in the acidic microenvironment caused by cellular activities, leading to increased supersaturation of the biological or physiological fluid. Further, precipitation of CO_3_-HAp together with other ions and organic molecules occurs [32] (Figure 1). It is believed that biological apatite consists of a mixed substituted CO_3_-HAp, in which CO_3_^2−^ ions are substituted for both PO_4_^3−^ ions (type B, the major form) and OH^−^ (type A, the inferior form) [33]. 

In other words, biological apatite is a calcium phosphate framework incorporated by various sorts of ions. Calcium, phosphorus and oxygen are the three major elements composing the said framework. It was believed that the calcium/phosphorus (Ca/P) ratio of biological apatite was either lower than or close to that of stoichiometric HAp, namely, 1.67 [15, 29]. However, it was also reported that the Ca/P ratio could be even higher than the latter [5, 11, 12]. This may be ascribed to the difference in the treatment methods and conditions [14], the raw materials, and the test methods [12], as well as the detection error. Besides, carbon, sodium, potassium, fluorine, magnesium, aluminum, strontium, chloride, and some trace elements are also detected as incorporated ions in the biological crystals [29], although their content may vary among samples and sources, and the copresence of all these elements may not be always found. Such ionic substitutions could cause the difficulty in the measurement of the chemical compositions of biological apatite. Additionally, the dilemma in the determination of the chemical compositions is also worsened by ionic absorptions. For each crystal, these two effects may be distinctive, for its microenvironment *in vivo* may be unique. The location of absorbed trace elements can be hardly determined, that is, in the inorganic or the organic tissue of bones [34]. Furthermore, the incorporation and deposition of ions may increase with the age of crystals (namely, the elapsed time between the initial deposition of the crystal and its removal from the tissue), leading to a more complicated situation in the chemical compositions of biological apatite. In this case, the content of each element, especially the amount of chemical groups including OH^−^, CO_3_^2−^, PO_4_^3−^, and HPO_4_^2−^, in biological apatite may be substantially different from that in synthetic pure HAp. 

Biological apatite shows its unique chemical compositions in two aspects, that is, the lacking of anticipatory hydroxyl group and the existence of HPO_4_^2−^ [34]. For hydroxyl group, it was reported that only a few percentage of the predicated concentration was detected in bone [35]. This was mainly explained by a charged compensation, which was initially caused by multiple ionic substitutions occurring in the lattice of biological apatite much often. The existence of HPO_4_^2−^ could be resulted from ionic substitutions as well. Moreover, it may also be ascribed to the hydrolysis of PO_4_^3−^ in solid phase [34]. However, up to now, it is nearly impossible to accurately measure the amount of such chemical groups including OH^−^ and HPO_4_^2−^ due to the insufficient accuracy of analytical techniques [34]. This situation is worsened by the existence of a little known hydrated layer on the surfaces of the synthetic apatite crystals. The mineral ions consisting of such a layer were detected on the surfaces of biological apatite in nonapatitic arrays [36]. This layer is very difficult to be identified since the spectroscopic data of synthetic apatites have been found to be much more sensitive to partial and complete drying [34]. Similarly, the functional groups mentioned above can also be readily influenced by external factors such as temperature, pH, inorganic solvent, and ionic effect. For instance, the apatite could lose carbonate groups after being sintered up to 850°C for 2 hours [5]. Inorganic solvent such as acid, even pure water, may cause partial dissolution of biological apatite crystals as well [13].

### 2.2. Crystal Morphology and Structure

The crystal structure of synthetic HAp was reported long ago [27], and most of the basic knowledge of the crystal structure came from single crystal X-ray diffraction (XRD) and neutron diffraction studies. However, for biological apatite, the analysis of single crystal structures had not been possible because of the absence of suitable single crystals for study [29]. Nevertheless, it was reported that the isolated crystals from natural bones were poorly crystalline apatite, similar to powdered intact bone from which they were originated [13]. Unfortunately, no crystal lattice parameters were stated in that study. In some subsequent studies, polycrystalline parameters were reported. The parameters of biological HAp prepared from bovine bone by sintering to 700°C were *a* = *b* = 9.429 Å, *c* = 6.885 Å [37]. By contrast, the cell parameters of enamel in human and shark teeth were stated as *a* = *b* = 9.445 Å, *c* = 6.833 Å and *a* = *b* = 9.377 Å, *c* = 6.881 Å, respectively [31]. 

As to the shape of bone mineral crystals, it was reported that the crystals from bone sources were rod-like or needle-like in nanoscale [5, 38]. However, some studies insisted that the original biological nanocrystals derived from various bones were in a similar shape of thin plate with wrinkled edges. It was also argued that the so-called rod-like or needle-like shape resulted from a special observation angle of the crystals as well as the probable transformation caused by heat treatment [8, 11, 13, 39]. On the other hand, the crystal size of biological apatite had a great variety among reports and sources, from several nm to more than 100 nm [5, 8, 11, 13, 39]. Additionally, it was believed that crystal size was highly related to the age of animal, especially the age of crystal. The crystal harvested from young postnatal animal was reported to be shorter and thicker than that from mature individual [18]. The conception of crystal age was specially emphasized by Rey et al. [34]. It was stated that there was a strong correlation between crystal age and its chemical compositions as well as morphology. Even for the same individual and the same bone, the biological apatite crystals varied from each other in age, resulting in the difference of structural and compositional properties. 

**Figure 2:**
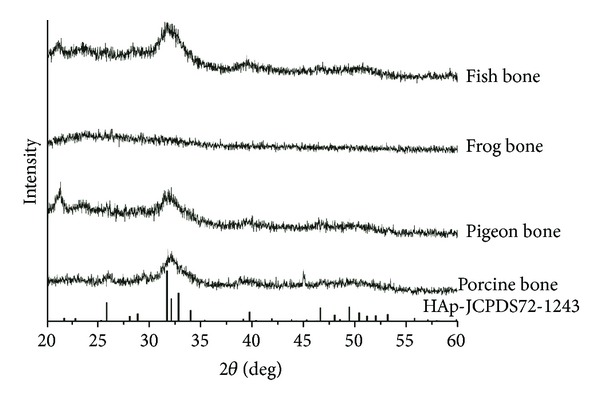
XRD patterns of animal bone-derived biological apatite: compared to the standard PDF card, the XRD patterns of the raw bones from various animals were hardly identified as HAp.

**Figure 3:**
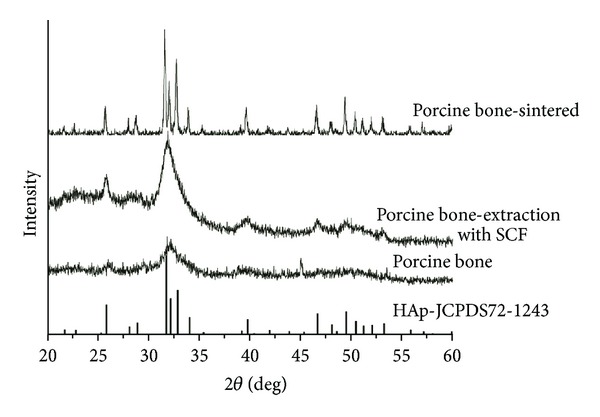
XRD patterns of biological apatite from porcine bone with different treatments: the XRD patterns of the raw porcine bone and those extracted with SCF were undistinguishable; the porcine bone sintered at 800°C for 2 h showed similar XRD patterns to the standard PDF card of HAp.

As a matter of fact, the crystal size and crystallinity can also be readily affected by treatment methods and conditions. The crystallinity of biological apatite was found to increase with the sintering temperature of bovine bone [16]. The effect of treatment approaches to crystallinity was found in a previous study [5], which could be also proven by one of our recent studies: series of animal fresh bones were examined with XRD (Figure 2). It was found that the XRD patterns of fresh bones were hard to be identified, indicating a low crystallinity for all these animal bones. By contrast, the porcine bone treated with supercritical fluid CO_2_ extraction (SCF, 30°C, 35 MPa, 2 h) showed a slightly distinguishable pattern with higher intensity (Figure 3). Still, it was difficult to identify the crystal nature with this pattern alone. Another set of porcine bone was sintered up to 800°C for 2 h, whose XRD pattern was fitted well with that of the standard PDF card of HAp (Figure 3). This demonstrated that a phase transformation together with crystal growth may have been completed during the sintering process, indicating that the crystalline characteristics can be easily affected by the treatment approaches. 

### 2.3. Dissolution Behavior and Solubility

As a matter of fact, the dissolution behavior and mechanism of biological apatite have been investigated since long time ago [40], and the solubility of biological apatite from bones and teeth has been extensively reviewed by Horvath [2]. That work drew a conclusion that comparable data of the solubility of biological apatite had been lacking, and there was a need for much more accurate solubility data. However, based upon a recent report, the solubility of bovine bone derived HAp (Bio-Oss) was significantly higher than that of synthetic HAp [6]. This may be ascribed to the negative effect of the higher crystallinity of synthetic HAp than that of biological apatite. Additionally, it was also partially explained as the inner effect of carbonate on the structure and dissolution behavior of biological apatite, similar to that of carbon dioxide to the dissolution of HAp reported before [20]. On the other hand, the presence of magnesium in the biological apatite may contribute to the increased solubility compared to synthetic HAp [6]. Nevertheless, Bio-Oss was made from bovine bone by the extraction of organics at around 300°C according to the data from its manufacturer, indicating that such solubility data may not be the same to that of the original biological apatite due to the probable transformation such as crystal growth and recrystallization that resulted from such a high temperature and some other unknown conditions in the preparation process. Consequently, rare consistent solubility data for biological apatite are available up to now.

**Figure 4:**
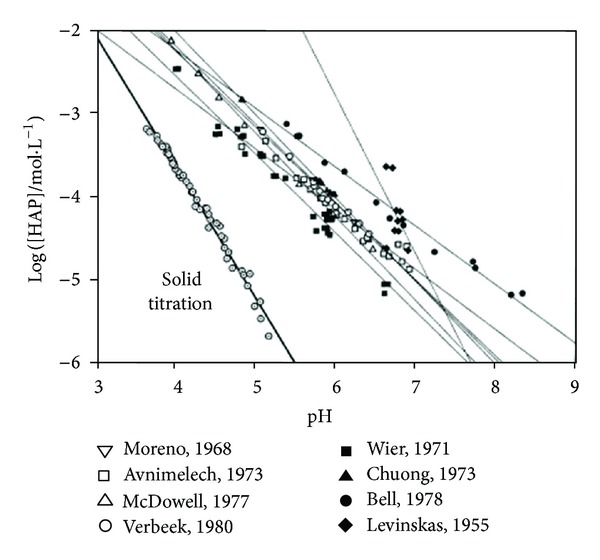
Solubility of hydroxyapatite with various methods [20].

Given the complexity of biological apatite that resulted from the foreign ionic incorporations, synthetic stoichiometric HAp, with relatively simple structure and composition, has been taken as a simulative model for the investigation and assessment of the solubility as well as the ionic effects to the dissolution process of biological apatite [20, 41–47]. Still, the dissolution of synthetic HAp appears much more complicated compared to that of simple salts since the former is incongruent. Several simultaneous parallel aqueous and surface reactions are involved in the dissolution process of HAp so that the classic thermodynamic theory could hardly explain the mechanism [48]. In this case, little agreement on the solubility and the definite dissolution mechanism has been achieved although numerous studies attempting to solve this problem have been carried out over the past few decades (Figure 4) [20, 46, 49, 50].

With respect to the research progress and discrepancy among different research methods about the solubility of HAp, it has been fully reviewed in some previous publications [20, 41], which could be summarized as follows.

Firstly, due to the incongruent dissolution of HAp and the complicated reactions in the dissolution system, it is inappropriate to determine its solubility and investigate the dissolution mechanism with the traditional methods [17] based upon excess addition of solid, long period of immersion, and the classic thermodynamic theory. The discrepancy of the reported solubility of HAp in the previous publications may be also caused by the differing compositions (including Ca/P ratio) of the samples and the contamination of other phases (such as OCP, DCPD, and TCP), or ions (carbonate, especially). 

Secondly, solid titration [20, 41], as a reliable, precise, and reproducible method, can be used in the determination of the solubility of HAp as well as some similar complexes [6, 42]. Compared with the traditional excess-addition approaches, solid titration is based upon the expectation of heterogeneous nucleation close to the point of saturation [51]. The solubility of HAp determined with solid titration was substantially lower than that previously reported [20, 41]. In the solubility isotherm of HAp, a change of the slope was found at pH ~3.9, which may indicate two different dissolution modules or phases at its two sides [41]. Precipitates at pH 3.2, 3.6, and 4.1 were collected and identified as deficient-calcium HAp, whose Ca/P ratios appeared to decrease with the pH value [41]. Similar results were obtained in subsequent studies [6, 47], which was inconsistent with a previous report in which DCPD was presumed to be the stable phase below the singular point of DCPD/HAp at pH 4.3 at 25°C [52]. 

Thirdly, the effects of CO_2_, strontium, and excess phosphate on the dissolution of HAp were investigated with solid titration as well. It was found that the solubility was increased by such ions to some extent [20, 42, 47], indicating that the biodegradability of biological apatite may be improved with such ionic incorporations.

Although the solubility of HAp has been fully investigated, some outstanding problems related to the dissolution mechanism and the effect of some other factors should be resolved in further studies, which is of significance to the understanding of biological apatite. On one hand, the probable phase or complex during the dissolution process of HAp needs to be further identified. In some research models, for example, the metastable theory, probable surface complex or phase was presumed or even “proven” indirectly [53]. However, little direct evidence such as XRD patterns and SEM images has been obtained, although both stoichiometric HAp [53] and deficient-calcium HAp [54] were reported in different studies. On the other hand, the effect of ions and molecules on the dissolution behavior of HAp needs further investigation. Many studies have been carried out to investigate the effect of different ions and molecules on the properties of HAp [42, 47, 55, 56]. Nevertheless, some controversy remains and needs to be clarified.

## 3. Preparation of Biological Apatite

Biological hard tissues such as bones [11, 14, 16, 37, 57] and teeth [58] are the main raw materials in the preparation of biological apatite. For the commercial bone grafting products, most of the bone-derived substitutes are from bovine bone. However, other animal bones such as porcine and canine bones show great similarity in terms of macro-/micro-structure, bone composition and remodeling to human bone (Table 1) [59], which could be alternatives for bone grafting materials. Moreover, the biological apatite from marine animals such as fishes has low crystallinity and high content of effective trace element such as strontium [60], indicating its potential as bone substitute as well. Nevertheless, to our knowledge, such rare animal bones have been used in the production of bone grafting materials. 

In some basic studies, various raw materials (Tables 2 and 3) and preparation approaches were reported. The common mechanism is to eliminate the organic components of the raw materials with various methods, leaving the inorganic apatite for investigation, examination, and application.

### 3.1. Thermal Treatment

Thermal treatment may be one of the most straightforward methods in order to eliminate the organic components of biological materials. It depends on the oxidation of organics at high temperature. Usually, it is carried out in a furnace or oven, where the organics are burnt out and the apatite are obtained. The conditions for this process including time, temperature, and atmosphere of sintering varied among previous studies (Table 2). A series of annealing temperature from 400°C to 1200°C was used to investigate the change of bone blocks in color, crystal characteristics, morphology, and composition [16]. In that study, it was found that the organic components of the bone blocks were removed completely by heating the samples up to 600–700°C for 2 h [16]. By contrast, it was reported that nano-sized crystals of biological apatite were obtained from a cortical bone of rat after sintering the raw material up to 600°C for 24 h, without any organic components residual [62]. Besides, 500°C and 700°C overnight were also applied in the preparation of biological apatite from bovine bones [37, 57], while the highest temperature reported was 1300°C for 2 h [14] or 45 min [8]. From all these studies, similar biological apatite was harvested although the heating temperature and period were inconsistent. This may be partially ascribed to the difference in the pretreatment (mainly chemical treatment) and oxygen condition as well as the size of samples. It can be assumed that lower temperature and shorter period might be required for the organic components elimination with some chemical pretreatments, sufficient oxygen, and smaller sample size. 

With a simple procedure, relatively low requirement in equipment and a low risk in disease transmission of the resultant, thermal treatment seems a feasible method for the preparation of biological apatite. The annealing temperature and period depend on the sample size, oxygen supply, and some possible pretreatments. For a bone block in one cubic centimeter, a temperature range between 600°C and 1000°C for 2 h may be enough to eliminate the organic components [16]. Meanwhile, a probable decomposition from HAp to *β*-TCP occurring at more than 1000°C could also be avoided under this condition [16].

**Figure 5:**
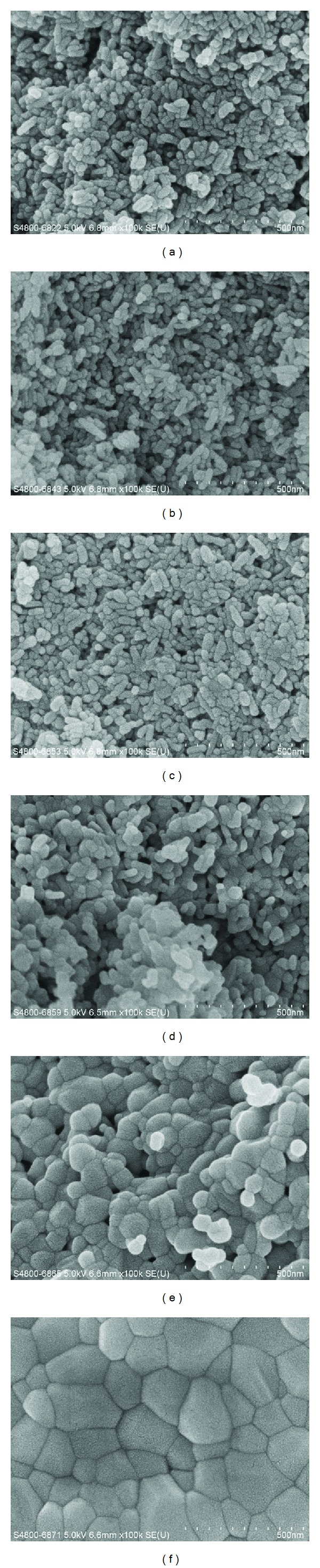
SEM images of synthetic HAp crystals ((a) HAp raw powder; (b)–(f) HAp-sintered at 600, 700, 800, 900, and 1000°C, resp.): HAp crystals became larger and aggregated when temperature >800°C.

Nevertheless, attention should be paid to the probable change occurring to the crystals of biological apatite from its original form. It is difficult to identify when and how many such a change occurs during the thermal treatment process, for the direct strong evidence was still lacking. However, upon an *X*-way investigation, it was found that the most significant change in the structure of biological apatite occurred between 600°C and 800°C, including a great increase of crystallinity and the disappearance of microstrain [67]. Besides, as to the possible effect of sintering temperature to the crystal characteristics, hint could also be drawn from some studies on synthetic HAp. It was reported that the morphology and crystal size of synthetic HAp were changed by heat treatment up to 650°C [68]. This was also observed in our recent study. HAp was synthesized with hydrothermal method and then sintered at 600, 700, 800, 900, and 1000°C for 2 h respectively. The resultants showed larger crystal size when the temperature was higher than 700°C, and aggregation of crystals was observed when annealing up to 800°C (Figure 5). Moreover, it was also observed that a thermal treatment up to 850°C for 1 hour resulted in a carbonate-free HAp [5]. It could be presumed that similar phenomenon might occur during the preparation process of biological apatite with thermal treatment as well. Consequently, the observed crystals may have larger size, changed morphology, and chemical compositions as well as different biological reactivity in comparison to their original form [61]. Hence, it is inappropriate to adopt thermal treatment as the preparation approach when investigating the original crystal characteristics of biological apatite. 

### 3.2. Hydrothermal Hydrolysis with Subcritical Water or Alkaline Solutions

Hydrothermal hydrolysis is a common method used for the synthesis of HAp [69–72]. However, it was less often used in the preparation of biological apatite. The detail was reported as follows. Ground bone powders were mixed with deionized water or alkaline solution (e.g., sodium hydroxide) at a certain solid/liquid ratio and put in a Teflon crucible, which was then placed in a cylindrical hydrothermal reactor made of stainless steel. This reactor was sealed tightly and heated in a silicon oil bath up to 250°C for 1 h or 5 h. Afterwards, the whole reactor was taken out and cooled down by quenching in a great deal of cold water. Finally, the solid product in the reactor was filtered, rinsed with deionized water, and dried [5]. The resultants were free of organic constituents, with maintained carbonate groups and varied crystallinity as well as crystal size.

Such a hydrothermal hydrolysis showed lower temperature in comparison to thermal treatment mentioned above. However, it is still hard to tell whether some unknown effects were caused by such a temperature with high pressure in the sealed reactor to the crystals or not. Nevertheless, partial dissolution of biological apatite crystals could have been unavoidably caused by the solutions used in the hydrolysis process [13], indicating that hydrothermal hydrolysis may not be viable in the preparation of biological apatite for an original-form investigation.

### 3.3. Chemical Treatment

Chemical treatment has been used attempting to remove the matrix of bones and teeth so as to obtain biological apatite. In particular, chloroform and methanol [13], hydrogen peroxide [14], acetone [5], and ether and its mixture with acetone [15, 37] were adopted as solvents in the elimination of fat. Besides, both alkali salt solutions such as sodium hypochlorite [64, 65] and sodium hydroxide [11, 17, 57] and nonaqueous hydrazine together with guanidine hydrochloride [13] were applied in the deproteinization process of raw bone samples. 

Chemical treatments are regarded as gentle approaches in the preparation of biological apatite, with which the phase transformation of crystals might be avoided and the carbonate groups could be reserved. Nevertheless, it was believed that the adopted chemicals may have an unclear influence on the structure and composition of the crystals [73], which may also affect the examination and application of the final material. In addition, such chemical treatments are usually ineffective and time-consuming. It may take hours or even days in order to remove the organic matrix. Even so, the result may not be so satisfactory, for organic components could be residual [39], especially when the bone samples are oversized and/or the reaction time is insufficient. Consequently, instead of being used independently, chemical treatment is more often taken as a pretreatment of other methods [13, 14, 17, 37, 57]. In this case, a chemical treatment may not be necessary as long as the other approaches can eliminate the organic components.

### 3.4. Supercritical CO_2_ Fluid Extraction

Supercritical fluid extraction was first invented and applied in some other fields rather than biomaterials science. Once a substance reaches the supercritical state, its physical property becomes an intermediate between the fluid and gas phases. In particular, the substance shows fluid-like density and gas-like diffusivity and viscosity, which enables it to dissolve nonpolar solids [66, 74]. Even so, by adding certain modifiers such as ethanol and propane, the solubility of both non-polar and polar solids could increase in a supercritical fluid. In particular, CO_2_ was chosen as the solvent due to its relatively low critical temperature and pressure, coupled with its wide availability, low cost, toxicity, and reactivity. Supercritical CO_2_ fluid has been used for the extraction of nonpolar and slightly polar species such as alkanes, alcohols, and fats [74]. 

**Figure 6:**
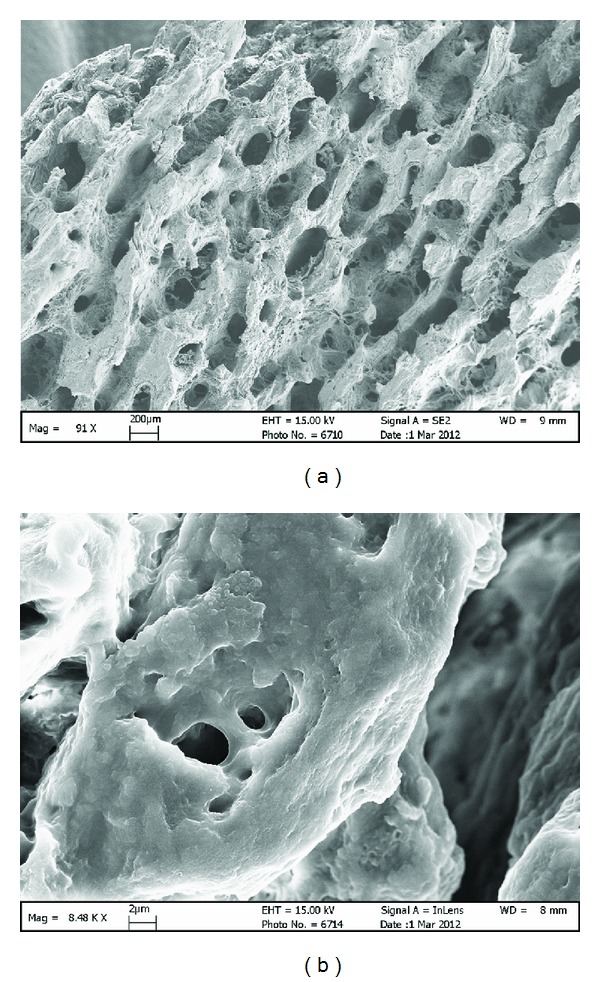
SEM images of porcine bone (supercritical CO_2_ fluid extraction at 30°C, 35 MPa, 2 h): (a) a porous interconnected bone structure was observed in a low power field; (b) most of the biological apatite crystals were embedded in the residual organic components.

With supercritical CO_2_ fluid (SCF), a porous interconnected framework that is composed of biological apatite and proteins could be obtained from animal bones at low temperature [75]. Such a resultant may be probably usable as a carrier or framework in bone engineering. However, it should be noted that most of the proteins like collagens cannot be extracted and would be remained in the bone samples [66] according to the extraction mechanism. In this case, most of the biological apatite crystals are embedded in the residual collagens and are hard to be observed (Figure 6). Thus, supercritical CO_2_ fluid extraction may be feasible in the preparation of biological apatite using as the framework of bone engineering or even bone substitute. However, sufficient single crystals of biological apatite could hardly be obtained for observation and examination with this method alone.

### 3.5. Low Power Plasma Ashing

A plasma ashing apparatus was introduced and described in detail in a previous report [76]. This system depends on the reaction between atomic gas (such as oxygen) and target substances (such as organics) to form only gases, that is, CO_2_, H_2_, and H_2_O vapor [77]. After 15 h or even longer reaction time, single crystals essentially free of organic constituents were harvested, with the assistance of hydrazine and intermittent ultrasonication [13]. By controlling the reaction conditions, the temperature of the whole reaction system could be lower than 30°C, preventing the probable effect of high temperature on the morphology, particle size, and crystallinity of the crystals [13, 39]. Besides, no aqueous solution was used during the preparation process of biological apatite crystals, so that the partial dissolution and recrystallization of crystals were avoided. 

Upon low power plasma ashing, biological apatite from various species was found to be poorly crystallized crystal with similar morphology, that is, long platelet crystal with wrinkled edges. No rod- or needle-like crystals were observed. Besides, the crystal size varied among different species, but in general the average crystal dimensions were much similar [39].

Given this, low power plasma ashing is probably a feasible method for the maintenance of the original characteristics of biological crystals. Nevertheless, as stated in Table 3, this method was mainly adopted in only one research group, and rare other related reports with this method were found, which might be resulted from the expensive equipment, a relatively complex procedure and long treatment period. Consequently, it seems that more evidence is needed to make this method as a convincing approach in the preparation of biological apatite.

In brief, as stated in Table 4, all approaches above could be applied in obtaining biological apatite. However, it seems that only low power plasma ashing was able to reserve the original characteristics of biological apatite, while either partial dissolution or transformation of the apatite crystals may be caused by the rest. By contrast, other methods are not able to maintain the original status of biological apatite for examination and assessment. However, both thermal treatment and hydrothermal hydrolysis could produce biological apatite for clinical application and reduce the risk of disease infection at the same time. Besides, chemical treatment could work as a supplementary method to other approaches, while supercritical CO_2_ fluid extraction may be adopted for the framework preparation in bone engineering by controlling the transmission of diseases.

## 4. Conclusion and Perspective

Together with synthetic HAp, biological apatite plays an important role in various fields. However, a comprehensive understanding of biological apatite has not been achieved due to its complicated basic properties. The consistent and comparable data for its chemical compositions, crystalline characteristics, and solubility were still lacking. Among the various approaches reviewed, there was not a standard or optimal method for the preparation of biological apatite. However, it seems that only low power plasma ashing can protect the apatite crystals from transformation, ionic incorporation, and partial dissolution, although more evidence is needed for convincing its reliability and feasibility. Given all above, more effort may be taken in the improvement of preparation method so as to achieve a better understanding of biological apatite and to improve the clinical performance of bone-based grafting materials.

## Figures and Tables

**Table 1:** Similarity between animal and human bone [59].

	Canine	Sheep/Goat	Pig	Rabbit
Macrostructure	++	+++	++	+
Microstructure	++	+	++	+
Bone composition	+++	++	+++	++
Bone remodeling	++	++	+++	+

+ least similar, ++ moderately similar, +++ most similar.

**Table 2:** Preparation of biological apatite with thermal treatment.

Authors	Raw material	Particle size	Pretreatment	Temperature (°C)	Annealing time	Crystal			
						Size	Morphology	Identity	Ca/P
Seo et al. [17]	Commercial bone ash powder	5–10 *μ*m	Immersion in NaOH	1000	1 h	0.5–1.0 *μ*m	Irregular	HAp + *α*-TCP	1.73
Janus et al. [11]	Porcine bone		Boiling; Leaching in NaOH	800 1200		70–700 nm		HAp (800°C) HAp + CaO (1200°C)	1.709 (800°C) 1.675 (1200°C)
Barakat et al. [5]	Bovine bone	<450 *μ*m	Washing with acetone	850	1 h	0.1–0.25 *μ*m		HAp	1.65
Seo and Lee [58]	Human teeth			900	1 h			HAp	1.63
Ooi et al. [16]	Bovine bone	10 ∗ 5 ∗ 5 mm^3^		400, 500, 600, 700, 800, 900, 1000, 1100, 1200	2 h			HAp (<1000°C) HAp + *β*-TCP (>1000°C)	>1.67
Rhee et al. [61]	Bovine bone		Degreasing and deproteinization	600, 800, 1000	3 h	50 nm (600°C) 1 *μ*m (1000°C)			
Murugan et al. [57]	Bovine bone		Degreasing; hydrothermal treatment in NaOH	500	Overnight	12 nm		HAp-CO_3_	
Danilchenko et al. [8]	Bovine bone	3 ∗ 5 ∗ 20 mm^3^ block and powder		100, 600, 700, 800, 900, 1000, 1100, 1300	45 min	Fast growth into big crystals (>200 nm, *t* > 600°C)		HAp (<700°C) HAp + CaO + MgO (>700°C)	
Kim et al. [14]	Porcine bone	15 ∗ 15 ∗ 15 mm^3^	Immersion in H_2_O_2_ and alcohol	1300	2 h			HAp + *β*-TCP (oxygen-poor environment) HAp (oxygen-rich environment)	1.49 (oxygen-poor environment) 1.66 (oxygen-rich environment)
Murugan et al. [37]	Bovine bone		Degreasing in acetone-ether mixture	700	Overnight			HAp	
Pan et al. [62]	Rat cortical bone		Immersion in H_2_O_2_ rinsed in deionized water and absolute ethanol and then dried, then being chilled in liquid nitrogen and pulverized	600	24 h	30–40 nm	Short rod	HAp-CO_3_ with trace Na and Mg	1.63 ± 0.04

**Table 3:** Preparation of biological apatite with plasma ashing.

Authors	Raw material	Sample size	Pretreatment	Ashing time	Crystal		
					Size	Morphology	Ca/P
Li et al. [63]	Goat bone powder		Degreasing in 2 : 1 chloroform and methanol mixture	15 h or more	8–10 nm in width	Plate-, rod-, needle-like	
Kuhn et al. [15]	Bovine tibia and femur	<75 *μ*m	Degreasing in 2 : 1 chloroform and methanol mixture	40 days			1.51 (younger cancellous bone) 1.61 (younger cortical bone) 1.58 (older cancellous bone) 1.64 (older cortical bone)
Kim et al. [39]	Chicken bone		Treated with hydrazine (10 mg/10 mL) for 12–24 h	15 h or more	103 nm (length) 68 nm (width)	Thin, wide, and relatively long rectangular plates	
Kim et al. [13]	Chicken, mouse, fish, and bovine bones	75–150 *μ*m	Extraction three times for 3 h at 4°C with chloroform and methanol mixture (2 : 1)	15 h or more	Bovine bone: 27.3 nm (length) 15.8 nm (width) Mouse: 21.2 nm (length) 12.0 nm (width) Chicken: 23.3 nm (length) 12.2 nm (width) Fish: 37.3 nm (length) 15.4 nm (width)	Thin plates	1.63 (bovine bone)
Tong et al. [18]	Bovine cortical bone	75–150 *μ*m	Extraction three times for 3 h at 4°C with chloroform and methanol mixture (2 : 1)	15 h or more	9 ± 3 nm (length) 6 ± 2 nm (width) 2.0 ± 1.2 nm (thickness)	Small platelets	
Eppell et al. [9]	Bovine cortical bone	75–150 *μ*m	Extraction three times for 3 h at 4°C with chloroform and methanol mixture (2 : 1)	15 h or more	12 ± 2 nm (length) 10 ± 2 nm (width) 0.61 ± 0.19 nm (thickness)	Plate-like	

**Table 4:** Comparison between preparation approaches for biological apatite.

Methods	Advantages	Disadvantages
Thermal treatment [5, 11, 14, 16, 17, 37, 57, 61]	Relatively low requirement for equipment Simple procedure Manageable conditions Low risk in disease infection Relatively low cost Large amount of resultant	Unpredictable effect of heating on apatite crystals Properties of resultant vary upon different sintering conditions

Chemical treatment [64, 65]	Possible reservation of the original crystal form and composition Avoidance of heating effect Supplementary role for other methods	Residual chemical and organic components Possible precipitation of amorphous Ca-P phases [34] Time consuming

Low power plasma ashing [13, 15, 63]	Possible reservation of the original crystal form and composition Avoidance of heating effect, partial dissolution, and recrystallization	Relatively complex procedure Time consuming Small amount of resultant Special equipment are required

Supercritical CO_2_ fluid extraction [66]	Relatively low cost Possible reservation of the original crystal form and composition Avoidance of heating effect, partial dissolution, and recrystallization Large amount of resultant	Time consuming Residual organic components Special equipment are required

Hydrothermal hydrolysis [5]	No residual organic components Simple procedure Manageable conditions	Special equipment are required Unpredictable effect of high pressure on apatite crystals Properties of resultant vary upon different hydrolysis conditions

## References

[1] Wenk HR, Heidelbach F (1999). Crystal alignment of carbonated apatite in bone and calcified tendon: results from quantitative texture analysis. *Bone*.

[2] Horvath AL (2006). Solubility of structurally complicated materials: II. Bone. *Journal of Physical and Chemical Reference Data*.

[3] Cannizzaro G, Felice P, Leone M, Viola P, Esposito M (2009). Early loading of implants in the atrophic posterior maxilla: lateral sinus lift with autogenous bone and Bio-Oss versus crestal mini sinus lift and 8-mm hydroxyapatite-coated implants. A randomised controlled clinical trial. *European Journal of Oral Implantology*.

[4] Stavropoulos A, Karring T (2010). Guided tissue regeneration combined with a deproteinized bovine bone mineral (Bio-Oss) in the treatment of intrabony periodontal defects: 6-year results from a randomized-controlled clinical trial. *Journal of Clinical Periodontology*.

[5] Barakat NAM, Khalil KA, Sheikh FA (2008). Physiochemical characterizations of hydroxyapatite extracted from bovine bones by three different methods: extraction of biologically desirable HAp. *Materials Science and Engineering C*.

[6] Zhuofan C, Baoxin H, Haobo P, Darvell BW (2009). Solubility of bovine-derived hydroxyapatite by solid titration, pH 3.5–5. *Crystal Growth and Design*.

[7] Daculsi G, Kerebel B (1977). Some ultrastructural aspects of biological apatite dissolution and possible role of dislocations. *Journal de Biologie Buccale*.

[8] Danilchenko SN, Koropov AV, Protsenko IY, Sulkio-Cleff B, Sukhodub LF (2006). Thermal behavior of biogenic apatite crystals in bone: an X-ray diffraction study. *Crystal Research and Technology*.

[9] Eppell SJ, Tong W, Katz JL, Kuhn L, Glimcher MJ (2001). Shape and size of isolated bone mineralites measured using atomic force microscopy. *Journal of Orthopaedic Research*.

[10] Jackson SA, Cartwright AG, Lewis D (1978). The morphology of bone mineral crystals. *Calcified Tissue International*.

[11] Janus AM, Faryna M, Haberko K, Rakowska A, Panz T (2008). Chemical and microstructural characterization of natural hydroxyapatite derived from pig bones. *Microchimica Acta*.

[12] Joschek S, Nies B, Krotz R, Göpferich A (2000). Chemical and physicochemical characterization of porous hydroxyapatite ceramics made of natural bone. *Biomaterials*.

[13] Kim HM, Rey C, Glimcher MJ (1995). Isolation of calcium-phosphate crystals of bone by non-aqueous methods at low temperature. *Journal of Bone and Mineral Research*.

[14] Kim SH, Shin JW, Park SA (2004). Chemical, structural properties, and osteoconductive effectiveness of bone block derived from porcine cancellous bone. *Journal of Biomedical Materials Research B*.

[15] Kuhn LT, Grynpas MD, Rey CC, Wu Y, Ackerman JL, Glimcher MJ (2008). A comparison of the physical and chemical differences between cancellous and cortical bovine bone mineral at two ages. *Calcified Tissue International*.

[16] Ooi CY, Hamdi M, Ramesh S (2007). Properties of hydroxyapatite produced by annealing of bovine bone. *Ceramics International*.

[17] Seo DS, Kim YG, Lee JK (2010). Sintering and dissolution of bone ash-derived hydroxyapatite. *Metals and Materials International*.

[18] Tong W, Glimcher MJ, Katz JL, Kuhn L, Eppell SJ (2003). Size and shape of mineralites in young bovine bone measured by atomic force microscopy. *Calcified Tissue International*.

[19] Wang XY, Zuo Y, Huang D, Hou X, Li Y (2010). Comparative study on inorganic composition and crystallographic properties of cortical and cancellous bone. *Biomedical and Environmental Sciences*.

[20] Chen ZF, Darvell BW, Leung VWH (2004). Hydroxyapatite solubility in simple inorganic solutions. *Archives of Oral Biology*.

[21] Misra V, Chaturvedi PK (2007). Plant uptake/bioavailability of heavy metals from the contaminated soil after treatment with humus soil and hydroxyapatite. *Environmental Monitoring and Assessment*.

[22] Yukna RA, Castellon P, Saenz-Nasr AM (2003). Evaluation of hard tissue replacement composite graft materials as ridge preservation/augmentation material in conjunction with immediate hydroxyapatite-coated dental implants. *Journal of Periodontology*.

[23] Lu KL, Meng XC, Zhang JX, Li X, Zhou M (2007). Inhibitory effect of synthetic nano-hydroxyapatite on dental caries. *Key Engineering Materials*.

[24] Palmer LC, Newcomb CJ, Kaltz SR, Spoerke ED, Stupp SI (2008). Biomimetic systems for hydroxyapatite mineralization inspired by bone and enamel. *Chemical Reviews*.

[25] Fernández-Pareja A, Hernández-Blanco E, Pérez-Maceda JM, Rubio VJR, Palazuelos JH, Dalmau JM (2007). Prevention of osteoporosis: four-year follow-up of a cohort of postmenopausal women treated with an ossein-hydroxyapatite compound. *Clinical Drug Investigation*.

[26] Uskoković V, Uskoković DP (2011). Nanosized hydroxyapatite and other calcium phosphates: chemistry of formation and application as drug and gene delivery agents. *Journal of Biomedical Materials Research B*.

[27] Posner AS, Perloff A, Diorio AF (1958). Refinement of the hydroxyapatite structure. *Acta Crystallographica*.

[28] Elliott JC, Mackie PE, Young RA (1973). Monoclinic hydroxyapatite. *Science*.

[29] Elliott JC (1994). *Structure and Chemistry of the Apatites and Other Calcium Orthophosphates*.

[30] Ma GB, Liu XY (2009). Hydroxyapatite: hexagonal or monoclinic?. *Crystal Growth and Design*.

[31] Vallet-Regí M (2008). *Biomimetic Nanoceramics in Clinical Use from Materials to Applications*.

[32] LeGeros RZ (2008). Calcium phosphate-based osteoinductive materials. *Chemical Reviews*.

[33] Kolmas J, Szwaja M, Kolodziejski W (2012). Solid-state NMR and IR characterization of commercial xenogeneic biomaterials used as bone substitutes. *Journal of Pharmaceutical and Biomedical Analysis*.

[34] Rey C, Combes C, Drouet C, Glimcher MJ (2009). Bone mineral: update on chemical composition and structure. *Osteoporosis International*.

[35] Pasteris JD, Wopenka B, Freeman JJ (2004). Lack of OH in nanocrystalline apatite as a function of degree of atomic order: implications for bone and biomaterials. *Biomaterials*.

[36] Lu HB, Campbell CT, Graham DJ, Ratner BD (2000). Surface characterization of hydroxyapatite and related calcium phosphates by XPS and TOF-SIMS. *Analytical Chemistry*.

[37] Murugan R, Kumar TSS, Rao KP (2002). Fluorinated bovine hydroxyapatite: preparation and characterization. *Materials Letters*.

[38] Arsenault AL (1988). Crystal-collagen relationships in calcified turkey leg tendons visualized by selected-area dark field electron microscopy. *Calcified Tissue International*.

[39] Kim HM, Rey C, Glimcher MJ (1996). X-ray diffraction, electron microscopy, and Fourier transform infrared spectroscopy of apatite crystals isolated from chicken and bovine calcified cartilage. *Calcified Tissue International*.

[40] Garnier P, Voegel JC (1976). Kinetic studies of synthetic and biological apatite dissolution. *Journal of Dental Research*.

[41] Pan HB, Darvell BW (2007). Solubility of hydroxyapatite by solid titration at pH 3-4. *Archives of Oral Biology*.

[42] Pan HB, Li ZY, Lam WM (2009). Solubility of strontium-substituted apatite by solid titration. *Acta Biomaterialia*.

[43] Kim YG, Seo DS, Lee JK (2008). Dissolution of synthetic and bovine bone-derived hydroxyapatites fabricated by hot-pressing. *Applied Surface Science*.

[44] Prakash KH, Kumar R, Ooi CP, Cheang P, Khor KA (2006). Apparent solubility of hydroxyapatite in aqueous medium and its influence on the morphology of nanocrystallites with precipitation temperature. *Langmuir*.

[45] Heslop DD, Bi Y, Baig AA, Higuchi WI (2004). Metastable equilibrium solubility behavior of carbonated apatite in the presence of solution strontium. *Calcified Tissue International*.

[46] Zhuang H, Baig AA, Fox JL (2000). Metastable equilibrium solubility behavior of carbonated apatites in the presence of solution fluoride. *Journal of Colloid and Interface Science*.

[47] Pan HB, Darvell BW (2010). Effect of carbonate on hydroxyapatite solubility. *Crystal Growth and Design*.

[48] Neuman WF, Mulryan BJ (1952). The surface chemistry of bone. VI. Recrystallization in vivo. *The Journal of Biological Chemistry*.

[49] Verbeek RMH, Steyaer H, Thun HP, Verbeek F (1980). Solubility of synthetic calcium hydroxyapatites. *Journal of the Chemical Society, Faraday Transactions 1*.

[50] Margolis HC, Moreno EC (1992). Kinetics of hydroxyapatite dissolution in acetic, lactic, and phosphoric acid solutions. *Calcified Tissue International*.

[51] Pan HB (2007). *Solubility of Calcium Phosphates and Related Oral Minerals by Solid Titration*.

[52] Tung MS, Chow LC, Brown WE (1985). Hydrolysis of dicalcium phosphate dihydrate in the presence or absence of calcium fluoride. *Journal of Dental Research*.

[53] Chhettry A, Wang ZR, Hsu J (1999). Metastable equilibrium solubility distribution of carbonated apatite as a function of solution composition. *Journal of Colloid and Interface Science*.

[54] Bengtsson Å, Shchukarev A, Persson P, Sjöberg S (2009). A solubility and surface complexation study of a non-stoichiometric hydroxyapatite. *Geochimica et Cosmochimica Acta*.

[55] Eslami H, Solati-Hashjin M, Tahriri M (2010). Effect of fluorine ion addition on structural, thermal, mechanical, solubility and biocompatibility characteristics of hydroxyapatite nanopowders. *Advances in Applied Ceramics*.

[56] Sprio S, Tampieri A, Landi E (2008). Physico-chemical properties and solubility behaviour of multi-substituted hydroxyapatite powders containing silicon. *Materials Science and Engineering C*.

[57] Murugan R, Ramakrishna S, Rao KP (2006). Nanoporous hydroxy-carbonate apatite scaffold made of natural bone. *Materials Letters*.

[58] Seo DS, Lee JK (2008). Dissolution of human teeth-derived hydroxyapatite. *Annals of Biomedical Engineering*.

[59] Pearce AI, Richards RG, Milz S, Schneider E, Pearce SG (2007). Animal models for implant biomaterial research in bone: a review. *European Cells and Materials*.

[60] Rosenthal HL, Eves MM, Cochran OA (1970). Common strontium concentrations of mineralized tissues from marine and sweet water animals. *Comparative Biochemistry and Physiology*.

[61] Rhee SH, Park HN, Seol YJ, Chung C, Han SH (2006). Effect of heat-treatment temperature on the osteoconductivity of the apatite derived from bovine bone. *Key Engineering Materials*.

[62] Pan HB, Li ZY, Wang T (2009). Nucleation of strontium-substituted apatite. *Crystal Growth and Design*.

[63] Li ZY, Lu WW, Deng LF (2010). The morphology and lattice structure of bone crystal after strontium treatment in goats. *Journal of Bone and Mineral Metabolism*.

[64] Su X, Sun K, Cui FZ, Landis WJ (2003). Organization of apatite crystals in human woven bone. *Bone*.

[65] Weiner S, Price PA (1986). Disaggregation of bone into crystals. *Calcified Tissue International*.

[66] Chen CF, Chang CS, Chen YP, Lin T, Su C, Lee S (2006). Applications of supercritical fluid in alloplastic bone graft: a novel method and in vitro tests. *Industrial and Engineering Chemistry Research*.

[67] Rogers KD, Daniels P (2002). An X-ray diffraction study of the effects of heat treatment on bone mineral microstructure. *Biomaterials*.

[68] Pang YX, Bao X (2003). Influence of temperature, ripening time and calcination on the morphology and crystallinity of hydroxyapatite nanoparticles. *Journal of the European Ceramic Society*.

[69] Ma MG (2012). Hierarchically nanostructured hydroxyapatite: hydrothermal synthesis, morphology control, growth mechanism, and biological activity. *International Journal of Nanomedicine*.

[70] Li K, Tjong SC (2011). Hydrothermal synthesis and biocompatibility of hydroxyapatite nanorods. *Journal of Nanoscience and Nanotechnology*.

[71] Klinkaewnarong J, Swatsitang E, Maensiri S (2011). Synthesis and characterization of high purity hydroxyapatite nanorods by hydrothermal technique. *Journal of Nanoscience and Nanotechnology*.

[72] Parthiban SP, Elayaraja K, Girija EK (2009). Preparation of thermally stable nanocrystalline hydroxyapatite by hydrothermal method. *Journal of Materials Science: Materials in Medicine*.

[73] Sakae T, Mishima H, Kozawa Y (1988). Changes in bovine dentin mineral with sodium hypochlorite treatment. *Journal of Dental Research*.

[74] Phelps CL, Smart NG, Wai CM (1996). Past, present, and possible future applications of supercritical fluid extraction technology. *Journal of Chemical Education*.

[75] Bi L, Li D, Liu M (2010). The influence of approaches for the purification of natural cancellous bone grafts: morphology, microstructure, composition, strength and biocompatibility study. *Materials Letters*.

[76] Patterson JE (1979). Oxygen plasma asher. *Analytical Chemistry*.

[77] Dai XJ (1996). Kinetic model of an RF discharge in oxygen. *Australian Journal of Physics*.

